# Higher Order Multiscale Finite Element Method for Heat Transfer Modeling

**DOI:** 10.3390/ma14143827

**Published:** 2021-07-08

**Authors:** Marek Klimczak, Witold Cecot

**Affiliations:** Faculty of Civil Engineering, Cracow University of Technology, Warszawska 24 Street, 31-155 Cracow, Poland; plcecot@cyf-kr.edu.pl

**Keywords:** heat transfer, multiscale finite-element method, homogenization

## Abstract

In this paper, we present a new approach to model the steady-state heat transfer in heterogeneous materials. The multiscale finite element method (MsFEM) is improved and used to solve this problem. MsFEM is a fast and flexible method for upscaling. Its numerical efficiency is based on the natural parallelization of the main computations and their further simplifications due to the numerical nature of the problem. The approach does not require the distinct separation of scales, which makes its applicability to the numerical modeling of the composites very broad. Our novelty relies on modifications to the standard higher-order shape functions, which are then applied to the steady-state heat transfer problem. To the best of our knowledge, MsFEM (based on the special shape function assessment) has not been previously used for an approximation order higher than *p* = 2, with the hierarchical shape functions applied and non-periodic domains, in this problem. Some numerical results are presented and compared with the standard direct finite-element solutions. The first test shows the performance of higher-order MsFEM for the asphalt concrete sample which is subject to heating. The second test is the challenging problem of metal foam analysis. The thermal conductivity of air and aluminum differ by several orders of magnitude, which is typically very difficult for the upscaling methods. A very good agreement between our upscaled and reference results was observed, together with a significant reduction in the number of degrees of freedom. The error analysis and the *p*-convergence of the method are also presented. The latter is studied in terms of both the number of degrees of freedom and the computational time.

## 1. Introduction

Numerical modeling of the heterogeneous materials is a very active research field [1,2,3,4,5,6,7]. This is due to the fact that such materials are widely used in many important industry branches, e.g., civil engineering [1,2,6,7], automotive engineering [3,4], aerospace engineering [8] and many others. The composite’s superior performance is due to its manifold mechanisms, among others:

- A specific composite type, e.g., laminate [9,10] or matrix-inclusion [1,5];
- The specific properties, shapes [11] and weight/volume ratios of the constituents;
- A very high adhesion between the constituents [5,11].

The major aspects, listed above, are analyzed at their respective scale, i.e., atomistic, micro-, meso- or macroscale, in order to optimize the overall composite performance at the higher scale. The design process of the new composites is enhanced with numerical analyses that are complementary to laboratory experimentsm and provide an evident speed-up. A number of numerical methods are used to model composites. The most frequent applications are based on the finite-element method [1,11], the finite-difference method [2], the discrete element method [12] and other methods [5,13]. In our research, we make use of the higher-order, finite-element method. In this paper, only the *p*-convergence is studied, i.e., we keep the same mesh and sequentially increase the approximation order *p* within all elements. However, in our previous studies [14], we presented the application of the automatic *hp*-adaptivity [15,16] coupled with the multiscale finite-element method. The automatic *hp*-adaptivity enhances the whole framework due to its expected exponential convergence. In this paper, the scope is narrower concerning this aspect. The benefits of the higher-order multiscale-finite element method with the modified hierarchical shape functions in the multiscale analysis are demonstrated in Section 4.

A direct numerical analysis of the composite at the heterogeneity scale is frequently infeasible or unnecessary [17,18]. The analysis itself would be challenging in the context of the computational resources, as well as time-consuming. When completed, the results would provide an amount of data that could be impractical to present and demanding to store. Thus, a variety of upscaling methods was used to reduce the computation cost with the simultaneous incorporation of the lower-scale information in analyses carried out at the higher scale. Extensive revisions of the most popular upscaling methods can be found, for instance, in [19,20,21,22]. In our research, we made use of the multiscale finite-element method (MsFEM) in a form developed in [14,18,23,24]. A concise description of this is provided in Section 3.1. The outline and the specific features of the method are provided therein. Selected other multiscale finite-element methods [19] and similar approaches used for heat transfer analysis (or Poisson equations in general) are also presented in Section 3.1.

We mainly use MsFEM due to its flexibility. In the case of the composites’ analysis, the method exhibits a very important advantage that should be stressed here. Namely, it is free of the assumption regarding the distinct separation of the bridged scales. In some applications (e.g., asphalt pavement), this feature is useful in effective multiscale analysis. This is due to the relationship between aggregate particle diameters and layer thicknesses.

The natural parallelization, without any special amendments, introduced to the independent problems solved within neighboring subdomains, guarantees a substantial speed-up to the computations. A number of observations, which are thoroughly discussed in Section 3.1, give rise to additional computational time savings, which facilitate MsFEM implementation.

The main novelty of this paper consists of the application of the higher-order, multiscale, finite-element method to the steady-state heat transfer problem. The approach is based on a modified shape function construction. The auxiliary boundary value problem was introduced, and used for the heat transfer problem. In our previous papers [14,18,23,24], we applied a higher-order MsFEM to other partial differential equations (PDEs), namely, to the elasticity and viscoelasticity.

In this paper, we demonstrate an effective MsFEM performance and also test its *p*-convergence in order to illustrate the benefits of the higher-order approximation in a steady-state heat transfer problem. It is additionally discussed in terms of the computational time.

Finally, the proposed method was tested on very challenging numerical problems with a large contrast in terms of its material parameters (several orders of magnitude).

Ensuring the effective and reliable heat transfer analysis of the composites is one of the challenging numerical problems. Multiscale methods are the common approach in this case, due to the reasons given in the above paragraph. State-of-the-art papers devoted to the review and classification of multiscale methods developed for the heat transfer problem are present in the literature [25,26,27]. The main distinction refers to the analysis scale [27]; therefore, one can distinguish:

- The microscale modeling using molecular dynamics (MD) with the motion analysis of every single atom/molecule in the domain [28,29];
- The particle-based mesoscopic modeling based on a coarse-grained analysis, e.g., Monte Carlo method, lattice Boltzmann method [30];
- The macroscale modeling with the assumption of the continuum of the domain [2,13,31,32].

In order to avoid the problem of the excessively large amount of the information obtained in the direct composite analyses at any scale, a number of hybrid and multiscale methods are used for the heat transfer problem [27,28,30,31]. They can be classified in a manifold manner. In [27], they are mainly grouped according to the scales they bridge, e.g., molecular dynamics—continuum description, molecular dynamics—particle-based description. Below, following the distinction used in [27], we briefly present the selected methods using a description of the respective resolution, in order to subsequently upscale this. However, it should be noted that the coupling of scales is not exclusively performed for two neighboring scales. For instance, a hierarchical bridging between several scales can be performed, as in [33]. Thus, the coupling possibilities are not limited to those presented in the next section.

In this paper, the focus is only on the macroscale modeling. Namely, at both bridged scales, the assumption of the continuum of the domain is used. The purpose of describing the methods referring to lower analysis scales is to place the multiscale, finite-element method among other approaches. In the case of micro- and mesoscale analysis, we arbitrarily selected the most representative methods. For an extensive review of these, we refer the reader to [27,28,30,31]. In the case of the macroscale modeling, the description is slightly different. We present several representative methods, as well as a group of approaches that share similar concepts, as the multiscale finite-element method used in this study.

### 1.1. Microscale Modeling

In molecular dynamics, we analyze the motion of every single atom. An atom’s motion is described by Newton’s second law and interactions between atoms are described by the potential functions [27,30]. In the case of large domains, this approach is very time-consuming and provides an excessive amount of information. Thus, the research interest was to simplify the modeling.

The first coupled MD-continuum analysis was described in [34], and referred to the flow in a channel. The whole domain was divided into an atomistic and continuum region with a hybrid solution interface (HSI), where both of the descriptions were valid. The development of the methodology presented in [34] was mainly associated with the numerical treatment of the atomistic region and its coupling with the continuum region [35] (*coupling by states*). A coupling strength parameter ξ was introduced, due to the constrained dynamics used to transfer the data at the HSI. The study on this parameter led to further improvements [36,37] in the methodology presented in [34]. In [38], these two regions (continuum and MD) were coupled, introducing the fluxes in the HSI (*coupling by fluxes*). The USHER algorithm was also proposed [39] for both types of coupling to conserve the overall energy, momentum and mass by manipulating the number of atoms at the HSI.

The methods presented above constitute a group of hybrid methods, which use two regions with a different description resolution. Although these approaches are not purely heat-transfer-oriented, their methodologies can also be applied to the leading problem of this paper and to other elliptic problems. Due to the necessity of partial MD analysis in the domain, they remain relatively computationally demanding.

In order to actually bridge the MD scale with the continuum scale, a number of methods that originated from the heterogeneous multiscale method (HMM) [40] were developed. The main analysis was carried out at the continuum level with the local transfer of information from the atomistic scale.

### 1.2. Mesoscale (Particle-Based) Modeling

Unlike in the molecular dynamics, where we analyze the motion of every single atom/molecule, mesoscale modeling is based on a coarse-grained analysis [26,27]. This approach can be regarded as a kind of microscale analysis upscaling. A group of molecules is represented by the computational particle, and the mechanisms of such particles’ evolution were assumed. The differences between the main representatives of this group consist of a description of the particle evolution mechanism. The macroscale description was based on the continuum assumption at the higher scale, and the microscopic description at the lower scale is discrete. The mesoscale modeling is situated between these, i.e., the description is discrete but the scale of analysis (spatial and temporal) is substantially reduced.

When the particle motion is characterized by a probabilistic description, the direct simulation Monte Carlo method (DSMC) was established [27,30]. When the hydrodynamic behavior of the particles was mainly modeled by the addition of the dissipative force, dissipative particle dynamics (DPD) are used. Two other main representatives of this approach are the lattice gas automata (LGA) and the lattice Boltzmann method (LBM), which are based on the simplifications introduced to the collision term in the Boltzmann equation [27].

For the multiscale techniques bridging the microscale and mesoscale modeling, one can look at, e.g., the methods transferring the velocities between the scales [41] (MD, DPD and the continuum) or their distributions [42].

### 1.3. Macroscale Modeling

As far as the heat transfer modeling is concerned, the continuum assumption is typically used. It is also the main scale of interest within this paper. The methods which are most frequently [27] used to numerically solve the heat transfer problem at this scale are the finite-element method (FEM), the finite-difference method (FDM), the finite-volume method (FVM) and a number of further modifications. In our research, we make use of the higher-order finite-element method due to its expected exponential convergence, especially when combined with the automatic *hp*-adaptivity, as mentioned in Section 1.

The methods used to bridge between lower observation scales and the continuum description were mentioned in Section 1.1 and Section 1.2. It is natural that the processes occurring at these scales affect the macroscale response. The scope of this paper is narrower. Namely, we are only interested in multiscale analysis at the continuum level. The constituent properties are assumed to be known (e.g., from the laboratory test), but we analyze the effective response of a composite.

The very basic engineering approach is made to assess the effective properties of the material (the conductivity, in this case) on the basis of its underlying microstructure. In further analysis, the material is numerically modeled as the homogeneous one. For instance, direct or inverse mixture rule is used for this purpose.

In the case of the periodic domains, a theoretical approach to the asymptotic homogenization [43,44] was primarily used. This is based on the auxiliary BVP solution for a unit cell. Practically, a number of such BVPs are solved for the increasing unit cell sizes. This version of the homogenization for the Poisson equation was used, e.g., in [45,46]. Unlike this method, the improved MsFEM used in this study does not require the periodicity of the domain. It needs an auxiliary BVP to be solved, but the subdomain (a coarse mesh element) used for this purpose is typically selected only once.

The most popular approach used in the numerical analyses is the computational homogenization [5,22], which is based on the representative volume element (RVE) approach. Using the auxiliary BVP solution for every RVE (typically associated with the Gauss integration point), the quantities at the macroresolution are assessed using the Hill–Mandel condition. It is also free of the assumption of domain periodicity. If the finite element method is used at both analysis levels, the approach is known as FE${}^{2}$ homogenization [47,48]. The advantage of computational homogenization is that we do not need to assume a constitutive equation at the macroresolution, but can transfer the tangent stiffness tensor from the lower scale. The main limitation to this is the separation of the scales’ condition in the RVE analysis. Namely, the ratio of the characteristic dimensions of the micro- and macroscale should not exceed 0.1. In some structures, this condition does not hold. The improved MsFEM, used in this study, does not require this condition to be fulfilled. The computational homogenization for the heat transfer problem is discussed, e.g., in [49]. In a recursive manner, macro- to microscale transitions are performed. The pointwise macroscale response is transferred to the lower scale in a form of the boundary conditions for the BVP solved in the RVE (and associated with this point). From this microscale level, the averaged quantities are transferred to the macroscale.

In [13,31], the multiscale seamless-domain method (SDM) is presented. It has some similarities to both the computational homogenization and the multiscale finite-element method used in this paper. The macroscale solution is sought at the so-called coarse-grained point. Its surrounding subdomain is discretized arbitrarily (regardless of the mesh generated for a subdomain associated with neighboring coarse-grained point). The solutions sought are interpolated within these subdomains. Subsequently, the final solution is obtained using the SDM scheme to “average” the solutions resulting from the overlapping subdomains. In MsFEM, discretizations for the neighboring subdomains need to be compatible. Moreover, MsFEM is the FE${}^{2}$ scheme, unlike the SDM or the computational homogenization, which can be employed for any numerical method at the microscale level.

For instance, using the proper orthogonal decomposition (POD) at the RVE level constitutes the FEPOD method [50]. Therein, POD is used to reduce the basis and to speed-up the computations.

MsFEM shares a similar substructuring concept to the superelements [51]. This is a smart way of obtaining the fine mesh solution, using static condensation and solving the reduced problem only for the nodes associated with the skeleton. Consequently, the discretizations within the neighboring superelements need to be compatible. Unlike this concept, MsFEM first delivers the upscaled coarse-mesh solution, which can be transferred elementwise to the corresponding fine meshes.

As was noted in [27], not all the multiscale frameworks can be easily classified using the distinction on the bridged scales. The multiscale finite-element method improved in this paper does not fall within the above-mentioned classification. Like, e.g., the computational homogenization [22], it uses the same material description level (continuum) at both bridged scales.

The similarities between the MsFEM presented in this paper and the heterogeneous multiscale method (HMH) [40] should be underlined, since, in both cases, the microscale coupling u and macroscale U variables can be performed mutually by the respective operators. The means of their assessment constitutes the main part of the aforementioned methods. In HMH, the underlying microstructure is taken into account during the integration of the respective entries of the macroscale stiffness matrix and load vector. MsFEM enables the effective computation of these quantities through the multiplication operations of the assembled (within a coarse element) fine-mesh quantities.

A short comment on the nomenclature is necessary. There is a variety of so-called multiscale methods. The MsFEM version we use is based on a special shape function concept. There are also other approaches with a similar name. In [52], for instance, a multiscale finite-element method, based on the asymptotic expansion, is presented in applications to a periodic microstructure.

For a comprehensive description of the other existing homogenization methods, we refer to [20,21,22].

The remaining part of this paper is organized as follows. Section 2 constitutes a brief description of the analyzed heat transfer problem. For the sake of clarity, we limit this paper to the steady-state heat-transfer problem. In Section 3 an outline of the developed MsFEM version is presented, together with the formulation of the boundary value problem used to assess the special shape functions. Additionally, some comments on the numerical implementation are provided. In Section 4, the numerical results are shown to illustrate the performance of the introduced method. Finally, in Section 5, the findings of this paper are recapitulated and discussed.

## 2. Problem Formulation

We selected the steady-state heat-transfer equation as the test problem for the developed version of MsFEM. Thus, we limit our problem to the resulting Poisson equation of the following form:

(1) $$\frac{\partial q_{x}}{\partial x}+\frac{\partial q_{y}}{\partial y}+\frac{\partial q_{x}}{\partial z}=f$$

Herein, *f* denotes the internal heat source; $q_{x}$, $q_{y}$ and $q_{z}$ denote the respective heat flux components. Using Fourier’s law, the latter can be expressed in terms of the temperature T(x,y,z), as:

(2) $$\begin{array}{c} q_{x}=-k\frac{\partial T}{\partial x}\\ q_{y}=-k\frac{\partial T}{\partial y}\\ q_{z}=-k\frac{\partial T}{\partial z} \end{array}$$

where *k* is the thermal conductivity coefficient. We limit this study to the isotropic material, so *k* is independent of direction. This assumption is not necessary; therefore, we used it to simplify the description. Moreover, the materials analyzed in this study exhibit such behavior. Inserting (2) to Equation (1), we obtain:

(3) $$-\frac{\partial }{\partial x}\left(k\frac{\partial T}{\partial x}\right)-\frac{\partial }{\partial y}\left(k\frac{\partial T}{\partial y}\right)-\frac{\partial }{\partial z}\left(k\frac{\partial T}{\partial z}\right)=f$$

for every subdomain with C${}^{1}$-continuous *k*, with both temperature and normal flux component continuity on the subdomain interfaces. In this paper, we use only two types of boundary conditions, i.e.,

- Dirichlet boundary conditions: $T\left(x,y,z\right)=T_{D}$ on $\partial \Omega_{D}$;
- Neumann boundary conditions: $q_{x}n_{x}+q_{y}n_{y}+q_{z}n_{z}=q_{N}$ on $\partial \Omega_{N}$ (n is the unit outward normal vector, $q_{N}$ denotes the heat flux across $\partial \Omega_{N}$).

## 3. Upscaling

In this chapter, the MsFEM outline is described and illustrated. Some comments on the implementation of the presented approach are also provided, to emphasize the potential benefits of its application in the numerical modeling of composites.

### 3.1. Idea

As was mentioned in the Introduction, MsFEM shares a similar concept to HMM [40]. Namely, there is a distinct hierarchy of scales. Let us name them the microscale and the macroscale. Unlike in the Introduction, they both refer to the continuum level, but the microscale resolves the heterogeneous material structure and the macroscale is the scale of the analyzed effective material response. The mappings between the micro- (u) and macroscale (U) degrees of freedom must be determined in the first step. As a result of the analysis, we obtain the macroscale solution, and the microscale one can be derived in the post-processing phase.

The construction of the prolongation operator P, which allows us to express the microscale solution in terms of the macroscale one (u=PU), as well as in the other way ($U=P^{T}u$), is the core of the argued methodology.

We proceed with two sets of compatible meshes. The whole domain is discretized at the macroresolution level with a coarse mesh. Then, each of its elements is substantially refined in order to capture all the heterogeneities at the microresolution level. In this manner, the coarse mesh is naturally compatible with this set of the corresponding fine meshes. In [14], we demonstrated the potential of *hp*-adaptivity application at the macroresolution level. Thus, this is also used here.

**Figure 1:**
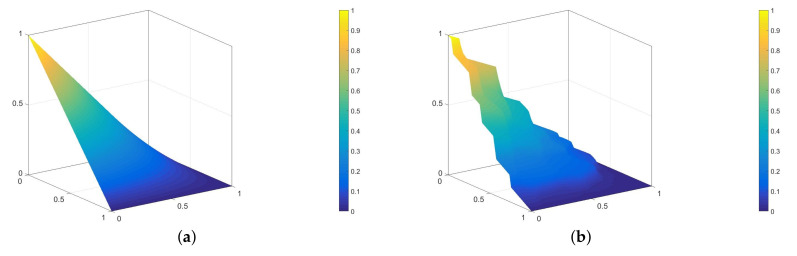
Exemplary bilinear standard (**a**) and modified (**b**) shape functions for the unit square coarse element.

In order to generate the prolongation operator P, we modify the standard coarse-element shape functions to account for the microstructure, by the solution of the auxiliary problem in coarse elements [14,23]. In Figure 1, we present a standard bilinear shape function and its modified counterpart, which was obtained as an exemplary composite. Knowledge of the operator P is used to compute the effective stiffness matrices and load vectors. Instead of integrating the modified shape functions over the coarse elements, we can immediately compute the effective stiffness matrix $K_{H}$ and load vector $f_{H}$ using the corresponding fine mesh quantities $K_{h}$ and $f_{h}$, which are assembled locally within a single coarse mesh element, since

(4) $$\begin{array}{ccc} K_{H} & \;=\;\; & P^{T}K_{h}P\\ f_{H} & \;=\; & P^{T}f_{h} \end{array}$$

For a number of tests on the special shape functions, we refer to [18]. Therein, MsFEM was used to solve the linear elasticity and viscoelasticity problems.

The general algorithm of the MsFEM application to the steady-state heat-transfer problem is analogous to the one presented in [18] for the linear elasticity. The difference, beside the different governing partial differential equation (PDE) to be solved (Equation (3)), consists of the other auxiliary boundary value problem (Equation (5)), which was consequently used for the special shape function assessment. This is marked in blue in Algorithm 1.

**Algorithm 1** Solve a heat transfer problem within a heterogeneous domain.**Require:** define the problem (heterogeneous domain and boundary conditions)
**Ensure:** a coarse mesh and an appropriate refinement of each coarse element
 **for**
*n*=1 to Nel
**do** {loop over coarse mesh elements}
  **for**
*m*=1 to *M*
**do** {loop over *n*-th element shape functions}
   solve local problem (5) in the *n*-th element for the *m*-th shape function
  **end for**
  compute $K_{H}$ and $f_{H}$ for the *n*-th element
 **end for**
 solve the coarse mesh problem using the effective matrices $K_{H}$ and vectors $f_{H}$


### 3.2. Formulation

The problem formulation for the assessment of the modified shape function is presented below. This is solved within a subdomain $\Omega_{s}$ (occupied by a single coarse mesh element) of the whole analyzed domain Ω. We need to solve problem (5) for every coarse-element shape function. The degrees of freedom obtained for the *m*-th standard shape function Ψ are the *m*-th column of the prolongation operator P. The problem presented below expresses the equality of the residuum of the solution (temperature) interpolant and residuum of the interpolated function $\Phi_{m}$.

*Given $\Psi_{m}$, which is a coarse mesh scalar-valued shape function (m = 1, …, M), we look for its scalar-valued counterpart $\Phi_{m}$, which is a discrete solution of the following Dirichlet boundary value problem*

(5) $$\left\{\begin{array}{c} \frac{\partial }{\partial x_{i}}\;\left(\;k\frac{\partial \Phi_{m}}{\partial x_{i}}\;\right)\;\;=-\frac{\partial^{2}}{\partial x_{i}^{2}}\Psi_{m}\;\forall i=\;1,\;2,\;3,\;x\in \Omega_{s}^{j}\subset \Omega_{s}\subset \Omega \\ \Phi_{m}={\hat{\Phi }}_{m}\;\mathrm{on}\;\partial \Omega_{s}\\ +\mathrm{interface}\mathrm{continuity}\mathrm{conditions} \end{array}\right.$$

*where k is the thermal conductivity of the material at a given location x, $\Omega_{s}^{j}$ denotes the j-th constituent of the composite*.

For the finite-element-method computations, we need a variational formulation, which is presented below.

*Find $\Phi_{m}\in V_{0}+\hat{\Phi }$ such that*

(6) $$\int_{\Omega }k\nabla v\cdot \nabla \Phi_{m}d\Omega =\int_{\Omega }v\Delta \Psi_{m}d\Omega \;\forall v\in V_{0}\;\;\;\mathrm{where}\;\;\;V_{0}=\left\{v\in H^{1}\left(\Omega_{s}\right):v=0\;\mathrm{on}\;\partial \Omega_{s}\right\}$$

This was obtained via the multiplication of (5) by the test function *v*, and the further integration of both equation sides over $\Omega_{s}$ (integrating by parts was also used at the left-hand side to transfer the derivative to the test function *v*). Practically, we additionally scaled the bubble functions in such a way that the extreme values were equal to 1.

**Figure 2:**
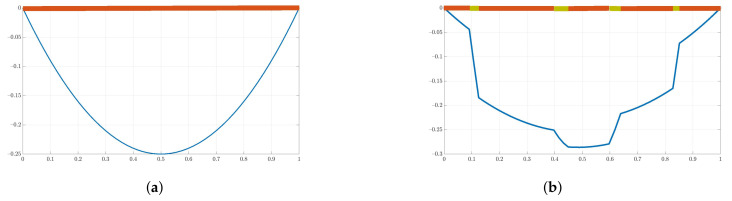
Exemplary “bubble” (**a**) standard and (**b**) modified shape functions for the unit 1D coarse element.

Definition of the Dirichlet boundary conditions ${\hat{\Phi }}_{m}$ needs special treatment regarding the dimensionality of the problem:

- In 1D, we only solve the reduced Equation (5) (i=1), obtaining the modified shape function. For the linear shape functions, we use 0 and 1 as the boundary conditions. For the “bubble” ones, ${\hat{\Phi }}_{m}$ is equal to zero. Exemplary standard and modified “bubble” shape functions for this case are shown in Figure 2. The horizontal thick lines represent the material distribution; thus, the standard shape function (Figure 2a) is the solution of problem (5) for the material with constant thermal conductivity in $\Omega_{s}^{1D}$. The solution presented in Figure 2b was obtained using 50 finite elements, which comply with the microstructure schematically marked with the horizontal line. The green material is characterized by a thermal conductivity 10 times larger than the other one;
- In 2D, we first solve the 1D problems along all necessary $\Omega_{s}^{2D}$ edges, as described above. Then, we use these solutions as ${\hat{\Phi }}_{m}$ for the Equation (5) solved for i=1,2. The exemplary result of such a two-step strategy is presented in Figure 1b;
- In 3D, we need to solve reduced problems (5) along the edges and, subsequently, within the faces of the domain. Finally, Equation (5) is solved with the Dirichlet boundary conditions resulting from the lower scales auxiliary computations. A number of 3D-modified shape function examples for the linear elasticity problem can be found in [14,18].

### 3.3. Implementation

We implemented [14,18] MsFEM for the linear elasticity and viscoelasticity problems. In this paper (Section 4), we present the results of the 2D analysis of the steady-state heat transfer using modified MsFEM.

To facilitate the multiscale computations, one can take advantage of two approaches, which are almost equivalent in terms of time saved:

- For the periodic heterogeneous domains, we compute the effective stiffness matrix $K_{H}$ once, and use it for every coarse mesh element—the effective load vectors $f_{H}$ are different in most cases;
- For non-periodic heterogeneous domains, we can parallelize the computations of the coarse-mesh-element matrices and vectors $K_{H}$ and $f_{H}$.

The second option is general, and the only time losses are due to the transfer of the local fine mesh computation ($K_{H}$ and $f_{H}$) to the global solver.

Another advantage of MsFEM, in the context of computational efficiency, is the possible low cost of Equation (5) solution. In fact, this is the main computational cost of the whole approach, since it has to be solved for every standard coarse-element shape function $\Psi_{m}$. However, it can be observed that Equation (5) leads to a system of algebraic equations with multiple right-hand sides. Let us denote the number of the functions $\Psi_{m}$ as *M*, and the time required for the solution of Equation (5) for a single $\Psi_{m}$ as $t_{\Psi }^{m}$. The overall computational time $T_{P}$ leading to the full operator P assessment is not, therefore, equal to $M\times t_{\Psi }^{m}$ but $T_{P}\ll M\times t_{\Psi }^{m}$, and it is of the order of $t_{\Psi }^{m}$. This is due to the fact that the computational time necessary to solve the system of linear algebraic equations with multiple right-hand sides does not significantly exceed the computational time used for a single system of linear algebraic equations.

In addition to the above discussion, one can observe that the matrix $K_{h}$, present in (4), is the same as the left-hand side of (5) for the arbitrary coarse element. Thus, the assembly can be performed once and is primarily used for Equation (5), and subsequently for Equation (4), which can also provide a further speed-up to the computations.

**Figure 3:**
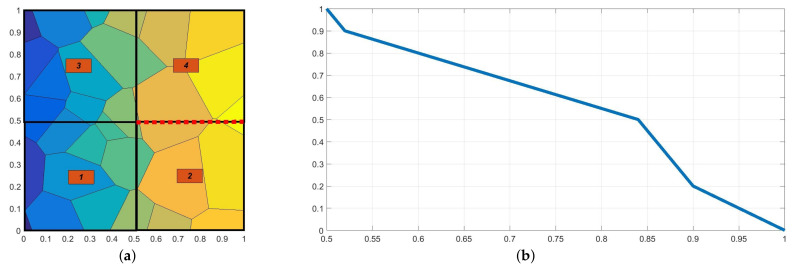
Exemplary shape function assessment: (**a**) analyzed domain—colors denote various thermal conductivities of the constituent, (**b**) modified linear shape function along the dotted edge.

Preserving the global continuity of the solution is necessary in the MsFEM workflow. In the case of the irregularly shaped constituents (see Figure 3a), the solution to Equation (5) along the edges of the coarse elements (denoted with numbers from 1 to 4) is straightforward. For instance, one of the modified linear shape functions for the common edge of elements 2 and 4 (marked with a red dotted line) is of the form shown in Figure 3b.

A situation where the coarse element edge coincides with different material interfaces needs special attention. When solving Equation (5) along an edge, thermal conductivity *k* needs to be used for a given x. Using different values for the common edge in a loop over coarse elements would not preserve the continuity of the sought function Φ within a 2D domain. To overcome this problem, we always take the average thermal conductivity at a given Gauss point ($\frac{k(x+\varepsilon )+k(x-\varepsilon )}{2}$, where ε is a small vector, orthogonal to the edge). This operation was performed to provide the continuity of the solution. If this was skipped, one could observe “jumps” in the solution of the interface (in the discussed situation). The accuracy would decrease with the increasing contrast between the constituents’ parameters.

## 4. Numerical Results

### 4.1. Asphalt Concrete

**Figure 4:**
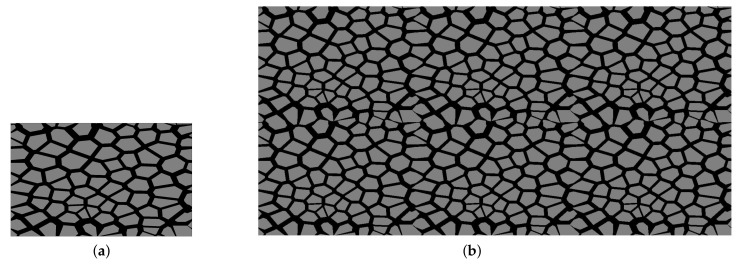
Synthetic asphalt concrete microstructure: (**a**) within a single coarse element, (**b**) within a whole domain.

In this test, we analyzed a sample with the dimensions 10 cm × 15 cm, made of asphalt concrete (AC), as shown in Figure 4b. AC is a standard asphalt mixture that can be applied for all asphalt layers constituting the flexible pavement structure. It comprises two main phases: mastic (the asphalt binder mixed with the filler) and the aggregate particles. The detailed procedure of the AC microstructure generation used for this test is presented in [24]. Given the prescribed gradation curve in asphalt concrete, we used the approach based on the shrunk Voronoi cells to generate a microstructure realizing this requirement. Using the method presented in [24], we generated a non-periodic microstructure in a coarse element with “periodic boundary conditions”, which enabled us to easily multiply this geometry. The temperature along the bottom edge was fixed and equal to 15 °C. The upper edge was subjected to heating with *q* = 30 W/m. The remaining edges were insulated (*q* = 0). Thermal conductivity was equal to 4 W/(mK) for the aggregate particles and 0.8 W/(mK) for the asphalt binder. In this test, we neglected the presence of the air voids.

**Figure 5:**
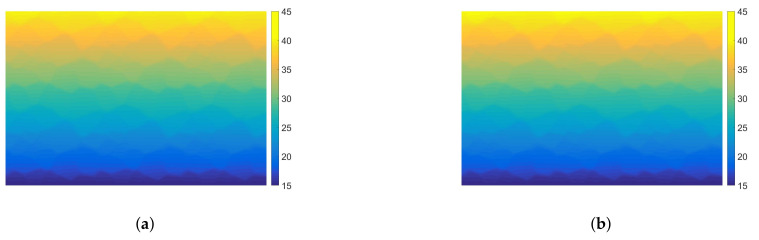
Temperature distribution [°C]: (**a**) direct FEM solution, (**b**) upscaled solution (plotted for *p* = 5).

**Figure 6:**
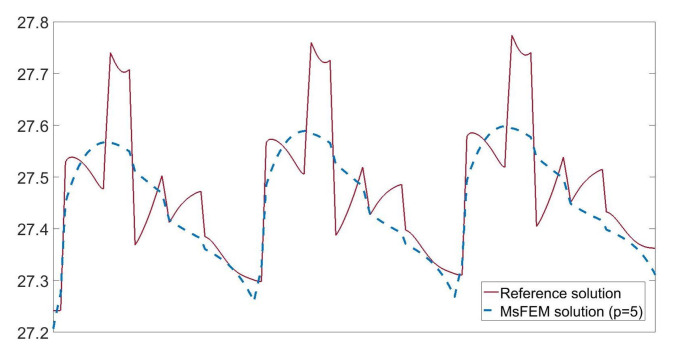
Temperature [°C] along the selected segment (at the half of the specimen height).

**Figure 7:**
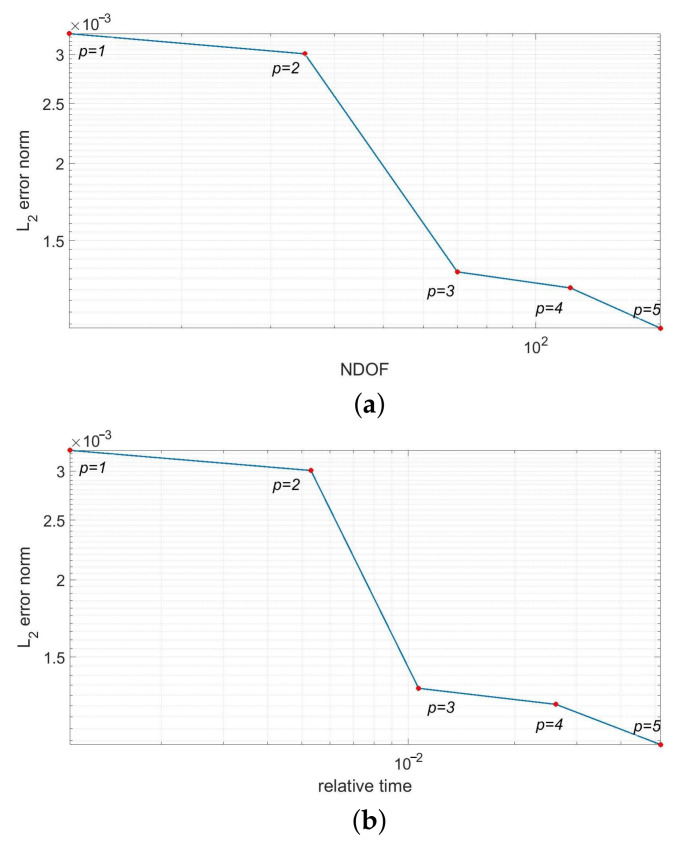
Convergence test for the increasing approximation orders at the macroscale (logarithmic scale) w.r.t. (**a**) number of degrees of freedom and (**b**) relative time (the ratio of the computational time used for the upscaled and reference solutions).

In Figure 5, we present the temperature distributions obtained using direct FEM analysis (*p* = 1) and MsFEM (*p* = 5). The corresponding cross-section plots, performed at half the specimen height, are shown in Figure 6. The modified shape functions for the coarse elements are similar to those presented in Figure 1b. The temperature distributions for the lower approximation orders, used at the macroscale, were skipped for the sake of brevity. They are visually indistinguishable from the solution presented in Figure 5b. Instead, the convergence plots are presented in Figure 7. They illustrate the applicability of MsFEM with respect to both the number of degrees of freedom and computational time.

We measure the error using the following formula

(7) $$\frac{|T^{h}-T^{H}|}{|T^{h}|}$$

where $T^{h}$ is the value of temperature at a given position obtained using the fine mesh and $T^{H}$ is the corresponding temperature obtained using the coarse mesh. By the fine mesh solution, we mean the reference solution plotted in Figure 5a. This was obtained using about 100,000 degrees of freedom.

The additional modeling error introduced by the upscaling is very small; even for the bilinear shape functions used at the macroresolution its norm, it is smaller than 0.4%. It should be noted that, in this case, we used only 12 degrees of freedom. For the approximation order equal to 5 at this scale, the number of degrees of freedom (NDOF) is equal to 176 and the error norm drops to about 0.1%. The number of coarse elements is kept constant and equal to 6. The reduction in degrees of freedom between the reference and upscaled solutions range from about 570 to about 8300. The results of this test confirm the *p*-convergence of MsFEM. The *h*-convergence was not studied in this paper. The focus was on the verification of the higher-order approximation applicability at the macroscale. In our previous papers [14,23], we also tested this type of convergence for the elasticity problem.

In our academic code, we only implemented the approximation of the order *p*, equal up to 5, for the heat transfer problem. The code would have to be modified to numerically verify the further shape of the convergence plot for this example. It should be noted that the macroscale *p*-convergence is affected by the microscale, and the corresponding discretization at this scale. A theoretical example of p→∞ would be cumbersome for this method. A prohibitively expensive fine mesh would have to be generated in order to approximate, with linear shape functions (typically used at the microscale level), a macroscale shape function of a very high order. This would be the case even for a very simple geometry. Practically, the order *p* = 5 is very rarely exceeded in numerical applications.

### 4.2. Metal Foam

**Figure 8:**
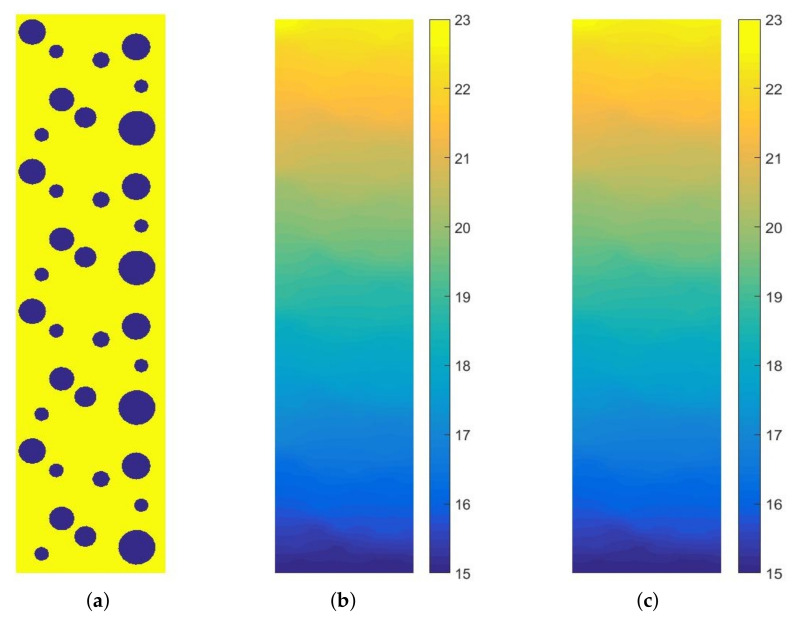
Material distribution (**a**) and temperature maps [°C]: (**b**) direct FEM solution, (**c**) upscaled solution (plotted for *p* = 5).

**Figure 9:**
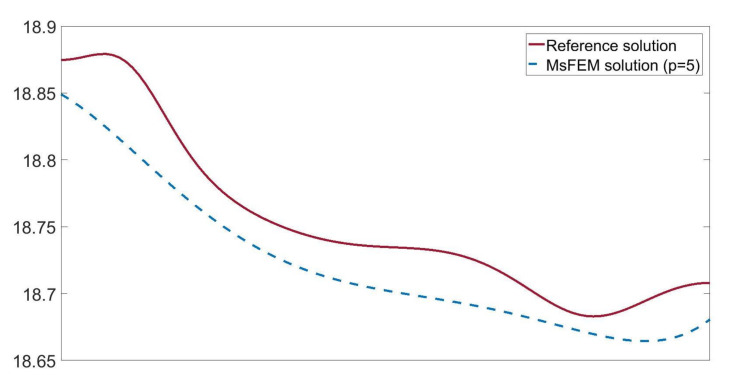
Temperature [°C] along the selected segment (at the half of the specimen height).

In the second test, we present the application of MsFEM in steady-state heat-transfer analysis of the metal foam. This lightweight material is used in many industry branches. In the context of the numerical modeling of heat transfer, the metal foam, analyzed with the application of any upscaling methods, can be regarded as a very challenging problem. This is due to the large difference between the constituents’ thermal conductivity. In this test, we analyzed the idealized aluminum foam sample with a matrix thermal conductivity equal to 236 W/(mK) and the thermal conductivity of the air was set as 0.0262 W/(mK). The rectangular sample of dimensions 2 cm × 8 cm was heated along the upper edge with *q* = 300 W/m. Along the bottom edge, the temperature is equal to 15 °C and the remaining edges are insulated. The material distribution, as well as the reference and upscaled solutions, are presented in Figure 8. The corresponding cross-section plots, performed at half of the specimen height, are shown in Figure 9. The air voids are modeled as circles with a random radii distribution.

**Figure 10:**
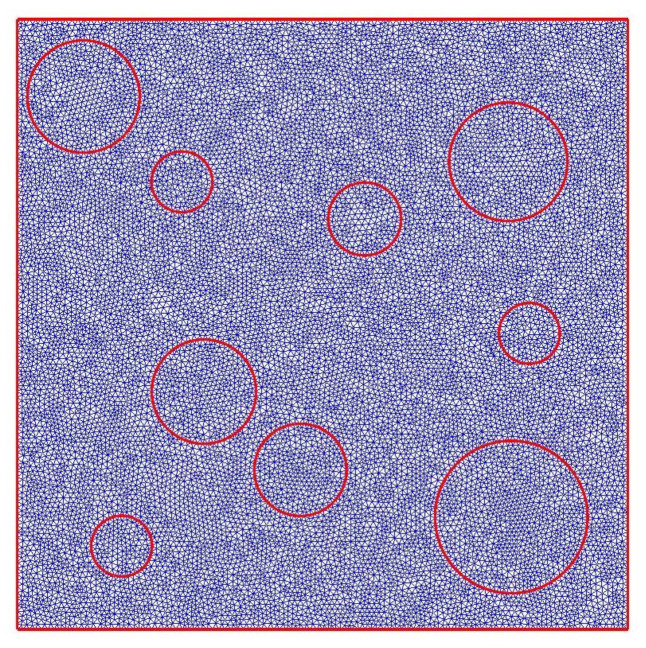
Fine mesh within a coarse element.

In this test, the reference solution was obtained using more than 69,000 degrees of freedom. The fine mesh within a single coarse element is shown in Figure 10.

**Figure 11:**
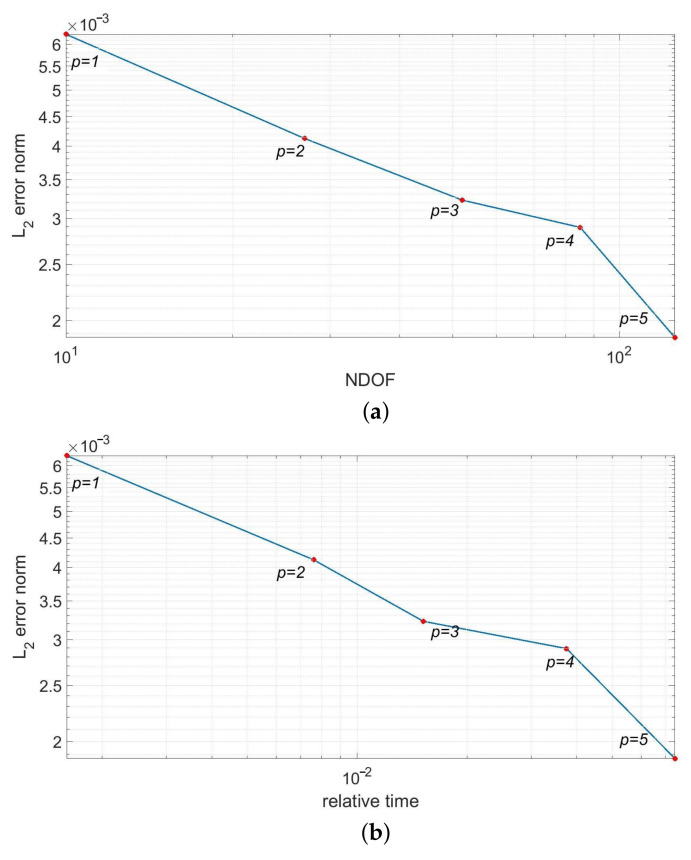
Convergence test for the increasing approximation orders at the macroscale (logarithmic scale) w.r.t. (**a**) number of degrees of freedom and (**b**) relative time (the ratio of the computational time used for the upscaled and reference solutions).

The upscaled solutions were obtained using 10 ÷ 126 degrees of freedom for the approximation order *p* = 1 ÷ 5, consecutively. The number of coarse elements is equal to 4. In Figure 11, we present the error convergence plots for the increasing approximation orders used at the macroscale. The convergence is demonstrated with respect to both the number of degrees of freedom and the computational time.

In this test, the $L_{2}$ norm of error (see Figure 11), measured according to 7, is equal to about 1.6% for *p* = 1 at the macroscale, and drops below 1.2% for the approximation order *p* = 5. The reduction in the number of degrees of freedom spans from about 550 (*p* = 5) to about 6900 (*p* = 1).

**Figure 12:**
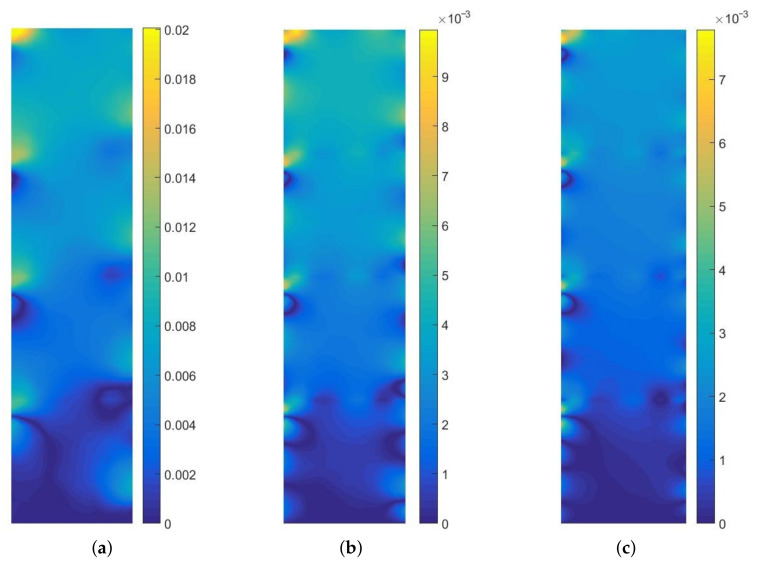
Error distribution for the increasing approximation order: (**a**) *p* = 1, (**b**) *p* = 3, (**c**) *p* = 5, consecutively.

In Figure 12, we present the upscaling error distribution for this test (for *p* = 1, 3, 5). We compute the error according to Formula (7). The differences span from about 0.76% (*p* = 5) to about 2% (*p* = 1). These discrepancies are acceptable from the engineering perspective. The character of error distribution around the air voids closest to the boundary is the result of a small number of finite elements, generated between this boundary and the void (see Figure 10).

Both convergence plots were created using the logarithmic scale to demonstrate the impact of the higher-order approximation at the macroscale on the result correctness. Technically, the comparisons between the reference and upscaled solutions are justified, since the mesh was generated as follows. First, the mesh was generated for a single coarse element. Then, it was copied several times to model the whole domain. In this manner, we compare the solutions obtained using meshes of the same densities.

A short discussion regarding the error convergence plots shown in Figure 7b and Figure 11b is necessary. Compared to the NDOF reduction in the upscaled solution, the observed speed-up is not equally impressive. This is due to the additional time necessary for the prolongation operator P assessment. It should be noted that this is highly affected by the implementation itself, as well as the discretizations at both scales. Moreover, MsFEM and other upscaling methods are addressed for problems, where the direct solutions are prohibitively computationally expensive or unfeasible to obtain. In this latter case, the computational time is less important than the ability to obtain the solution.

## 5. Concluding Remarks

In this paper, we proposed a higher-order MsFEM for the steady-state heat-transfer problem. The main novelty consists of the modified shape function assessment for this problem, presented in Section 3.2, and numerical confirmation of the upscaled solution *p*-convergence for a very large material contrast. We demonstrated the convergence of this method with respect to both the number of degrees of freedom and the computational time.

The proposed approach was verified on two tests, which were performed for the realistic materials. The first presents the application of MsFEM to the steady-state heat-transfer analysis of the asphalt concrete sample. In this example, the focus was on the method performance for a non-trivial microstructure, and there was not a large difference between the constituent thermal conductivity. The second test presented the application of MsFEM to a very challenging numerical problem. The steady-state heat transfer analysis of the aluminum foam was presented. In order to illustrate the superiority of the method, the air voids were included in the numerical modeling. Its presence implies the necessity of special composite modeling. Namely, we deal with a material with constituent parameters varying by several orders of magnitude. This is a very difficult problem for any upscaling method.

In both of the presented tests, a large reduction in the degrees of freedom was observed for the upscaled solutions. Compared to the reference fine mesh solutions, this was equal to 570 ÷ 8300 (asphalt concrete sample) and 550 ÷ 6900 (metal foam sample). It should be noted that, even for the harder test (metal foam) and the bilinear approximation, the maximum error is equal to about 2%. In both tests, the *p*-convergence was observed. Considering the large disproportion of the material parameters and the obtained NDOF reduction, this result is acceptable.

In terms of the computational time, the applicability of MsFEM was also demonstrated. The relative time, i.e., the ratio of the computational time necessary for the upscaled and reference solutions, is much smaller than the unity for both tests. The possible speed-up is highly affected by the implementation and discretizations at both scales. Thus, this can be increased. However, this was not the focus of this study.

Our further research effort is to extend the proposed framework to thermoelastic analysis. Such an approach is necessary, e.g., in the context of asphalt concrete modeling, where the thermal effects are very significant.

## Data Availability

Data sharing is not applicable.
